# Fish bone perforation mimicking colon cancer: A case report

**DOI:** 10.4102/sajr.v24i1.1885

**Published:** 2020-09-29

**Authors:** Thokozani Sibanda, Pria Pakkiri, Anne Ndlovu

**Affiliations:** 1 Department of Radiology, Baines Imaging Group, Harare, Zimbabwe; 2 Department of Pathology, Baines Pathology, Harare, Zimbabwe

**Keywords:** bowel perforation, fish bone, hemicolectomy, colonic malignancy, abscess, computed tomography

## Abstract

Most patients who ingest fish bones do not develop any complications. The small proportion of patients who do complicate, present with non-specific symptoms. A 64-year-old female patient presented with a 2-month history of abdominal pain. Following clinical evaluation and computed tomography scan of the abdomen, a provisional diagnosis of colon cancer was made. Histology of the resected bowel at hemicolectomy demonstrated a perforation by fish bone with an associated abscess. The case illustrates how fish bone perforation may mislead unsuspecting clinicians and may be misdiagnosed as colonic cancer.

## Introduction

Fish bone ingestion is a common problem, particularly in populations who consume unfilleted fish.^1^ It accounts for 84% of accidentally ingested foreign bodies. In most cases, accidentally ingested fish bones are eliminated from the gastrointestinal tract (GIT) without complications.^2^ Complications, however, do occur and these include entrapment in the upper aerodigestive tract, GIT perforation, sepsis and bowel obstruction.^1^

Bowel perforation is one of the serious complications, resulting mainly from the sharp or pointed edges of the fish bone.^3^ However, the incidence is less than 1%.^4^

We present a patient whose symptoms and clinical findings were commensurate with perforated colon cancer. A definitive diagnosis of fish bone perforation was made after hemicolectomy and histopathology analysis.

## Case report

A 64-year-old female patient presented to her general practitioner with a 2-month history of left upper quadrant pain. There had been no history of trauma or any specific precipitating event. No change in bowel habit was documented. On examination, she was a well-looking lady with mild left upper quadrant tenderness. No other significant findings were elicited at this time. Following a non-conclusive ultrasound, the general practitioner referred the patient to a general surgeon for further investigation.

**FIGURE 1:**
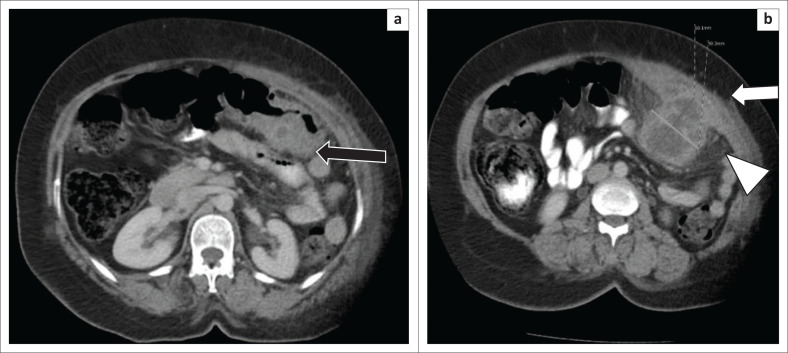
Selected axial computed tomography images demonstrated (a) an eccentric thickened wall of the distal transverse colon (black arrow) (b) with a focal, walled-off intra-abdominal fluid collection, extending from the wall of the transverse colon to the left anterior abdominal wall (white arrow). Invasion and thickening of the transversalis and rectus abdominis muscles was noted (white arrow). There was associated increased density and stranding in the pericolic fat and adjacent abdominal wall fat from the inflammation (arrowhead).

**FIGURE 2:**
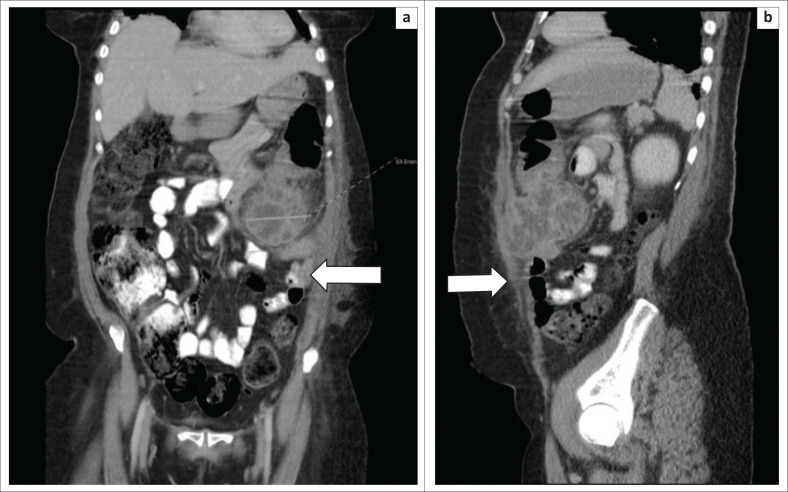
Selected coronal (a) and sagittal (b) images of the patient demonstrates the thick-walled distal transverse colon and associated walled-off collection closely related to normal appearing small bowel loops (white arrows).

After review of the history and examination, the general surgeon’s differentials were diverticulosis, inflammatory bowel disease and colon cancer. The patient was investigated with a computed tomography (CT) scan of the abdomen. Limited biochemical tests were ordered, mainly urea and electrolytes, which were essentially normal. Helical CT images were acquired from the lung bases to the symphysis pubis with multiplanar reformats, post intravenous and oral contrast administration (Figures 1 and 2).

Given the concern for a possible colonic adenocarcinoma, the patient was taken to theatre for surgical exploration. Drainage of the collection and a hemicolectomy was performed. Intraoperatively, a distal transverse colon ‘tumour’ with an abscess was identified. There were adhesions between the colon and jejunal loops in the left upper quadrant with an associated collection. The collection was drained and the resected bowel was sent for histological analysis. The patient had an uneventful recovery and was discharged home after a week.

**FIGURE 3:**
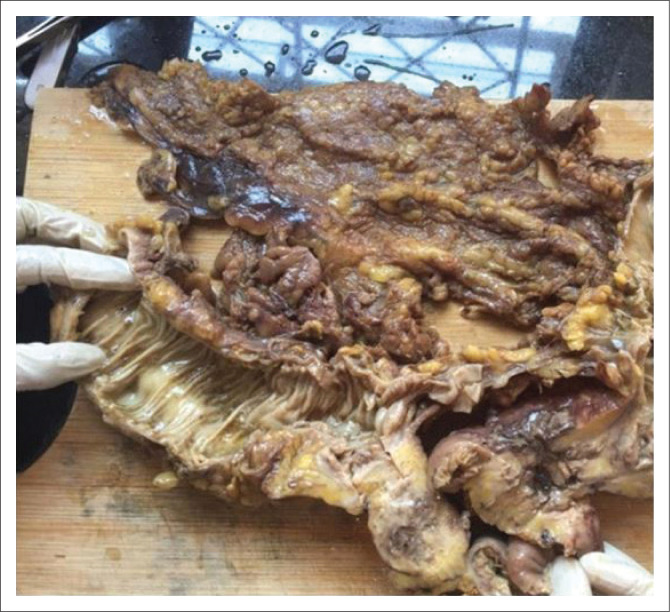
Multiple sections were sampled, all of which revealed soft tissue, consistent with inflammatory granulation tissue, and an abscess.

Upon opening the large bowel, gross pathology indicated a mass lesion (Figure 3). The cut surface was variegated and an abscess cavity was noted. There was no extension of the lesion into the adherent small bowel.

**FIGURE 4:**
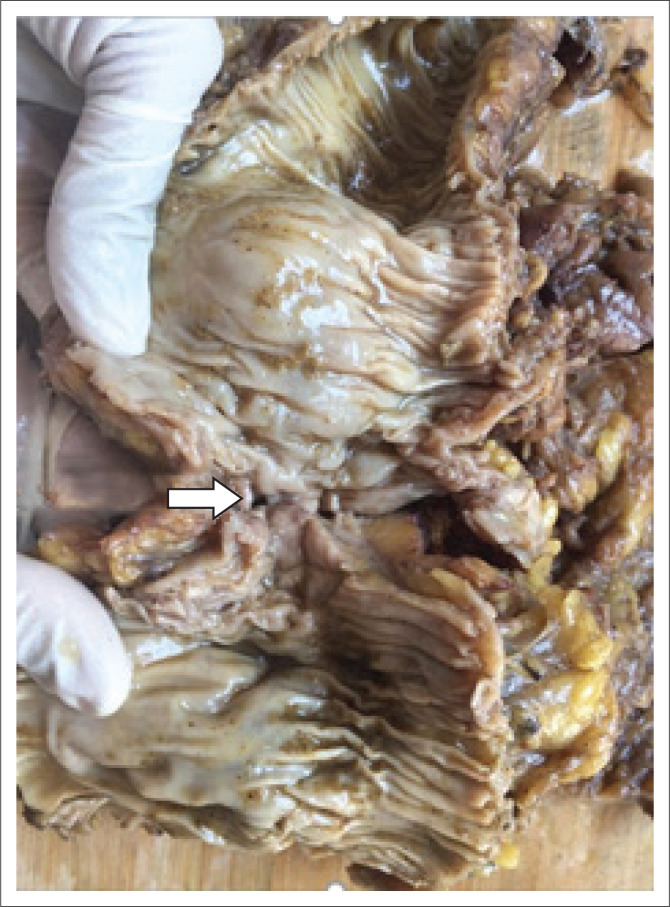
A small opening was identified in the bowel wall (arrow).

**FIGURE 5:**
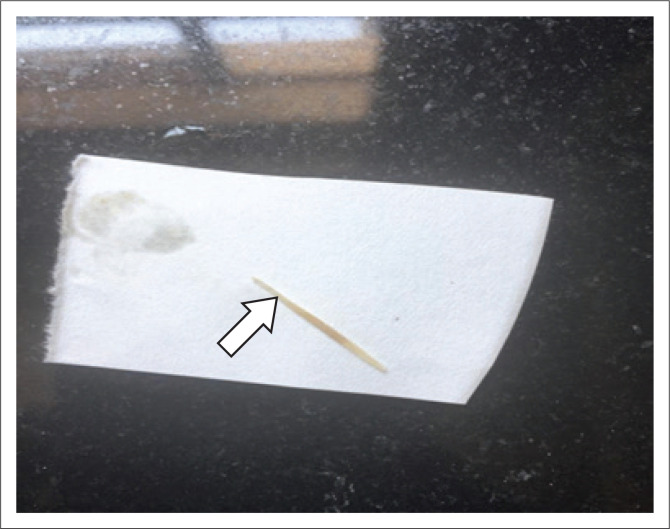
Further sectioning in this area revealed a 3 cm fish bone (arrow).

Since the histologic findings did not correlate with the clinical diagnosis, the specimen was re-examined to exclude a sampling error. A small opening was identified in the bowel wall and further sectioning revealed a fish bone (Figures 4 and 5).

### Ethical consideration

This article followed all ethical standards for carrying out research.

## Discussion

Fish bone perforation is a challenging diagnosis to make and frequently results in misdiagnosis. This is brought about by its bizarre presentation and the fact that patients may not recall ingestion of the fish bone. A time lag of weeks to months prior to presentation further compounds the problem.^1^

Perforation of the GIT distal to the oesophagus occurs in less than 1% of otherwise healthy patients.^4,5^ Patients with GIT perforation present with a myriad of symptoms, which include abdominal pain (which may be acute or chronic), GI haemorrhage, intestinal obstruction and ureteric colic.^3^ The preoperative diagnosis of fish bone perforation is confirmed in as little as 23% of cases. This can be attributed to the non-specific clinical presentation and the low sensitivity of radiological investigations.^6^

Plain film radiography plays a limited role in the detection of fish bones, especially below the hemidiaphragm. The sensitivity for detection of a fish bone in the aerodigestive tract is as low as 32%.^7^ False negatives are seen in up to 47% of cases.^8,9^ Sensitivity is influenced by the fish bone type as fish bones with a high calcium are more easily identified. Fish bones with less calcium may be obscured by soft tissue and fluid, thereby reducing sensitivity. Low kilovoltage supine radiographs demonstrate fish bones better than high kilovoltage techniques. In a study by Goh et al.,^3^ chest and abdominal radiographs failed to demonstrate the fish bone foreign body and pneumoperitoneum.^3^ In cases of fish bone related GIT perforation, pneumoperitoneum is rarely seen because there is limited passage of air and intraluminal fluid into the peritoneum as a result of the perforation being covered by omentum.^5^ With plain film radiography and CT, pneumoperitoneum is observed in 16% and 50% of cases, respectively.^3,4,10,11^

The imaging modality of choice is CT. A sensitivity of 100% has been shown in some previous studies. Computed tomography sensitivity is greatly influenced by a high index of suspicion. In the absence of a positive history of fish bone ingestion, there is the possibility of another structure such as a blood vessel being mistaken for a fish bone. Thin CT slice thickness and non-enhanced studies can better assist the radiologist to differentiate fish bones from other structures.^3,4^ A fish bone on CT may be seen as a linear calcified lesion. Associated CT findings at the site of perforation include: mucosal wall thickening of the bowel, intestinal obstruction, pericolic fat stranding and at times, there may be abscess formation. Inflammatory reaction can masquerade as a tumour. In the literature, cases of fish bone perforation have been misdiagnosed as appendicitis, pancreatic cancers, gastric submucosal tumour and Crohn’s disease.^12,13,14^

Cases of large bowel perforation in elderly patients may mimic colonic malignancy. In the absence of positive radiological identification of a foreign body, the non-specific symptoms and CT findings may simulate malignancy. In our 64-year-old patient, no fish bone was identified radiologically within the abnormally thickened colonic wall. Histology confirmed an inflammatory process at the area of perforation and no malignant cells were identified. A 3-cm fish bone was seen buried within the resected loops of bowel, confirming the diagnosis of complicated fish bone bowel perforation.

## Conclusion

Fish bone perforation in the lower GIT is an uncommon complication that may present with unusual symptoms and signs. Diagnosis requires a high index of suspicion from the treating clinicians. A thorough clinical history and radiological evaluation may help. This case highlights the fact that perforation by foreign bodies might have an indolent course rather than an acute presentation and highlights how fish bone perforation can mimic a malignancy.

## References

[1] Bathla G, Teo LL, Dhanda S Pictorial essay: Complications of a swallowed fish bone. Indian J Radiol Imag. 2011;21(1):63 10.4103/0971-3026.76061 PMC3056375 21431037

[2] Venkatesh SH, Karaddi NK CT findings of accidental fish bone ingestion and its complications. Diagn Interv Radiol. 2016;22(2):156 10.5152/dir.2015.15187 26714057 PMC4790067

[3] Goh BK, Tan YM, Lin SE, et al CT in the preoperative diagnosis of fish bone perforation of the gastrointestinal tract. Am J Roentgenol. 2006;187(3):710–714. 10.2214/AJR.05.0178 16928935

[4] Coulier B, Tancredi MH, Ramboux A Spiral CT and multidetector-row CT diagnosis of perforation of the small intestine caused by ingested foreign bodies. Eur Radiol. 2004;14(10):1918–1925. 10.1007/s00330-004-2430-1 15378256

[5] Choi Y, Kim G, Shim C, Kim D, Kim D Peritonitis with small bowel perforation caused by a fish bone in a healthy patient. World J Gastroenterol. 2014;20(6):1626 10.3748/wjg.v20.i6.1626 24587641 PMC3925874

[6] Sharma R, Padhy BP, Kumar S, Hareesh M, Suchithra GL Gastric perforation due to fish bone ingestion presenting as gastric outlet obstruction: A case report. Int Surg J. 2018;5(12):4081–4084. 10.18203/2349-2902.isj20185050

[7] Ngan JH, Fok PJ, Lai EC, Branicki FJ, Wong JO A prospective study on fish bone ingestion. Experience of 358 patients. Ann Surg. 1990;211(4):459 10.1097/00000658-199004000-00012 2322040 PMC1358032

[8] Kim HU Oroesophageal fish bone foreign body. Clin Endoscopy. 2016;49(4):318 10.5946/ce.2016.087 PMC4977739 27461891

[9] Qureshi TA, Awan MS, Hussain M, Wasif M Effectiveness of plain X-ray in detection of fish and chicken bone foreign body in upper aerodigestive tract. J Pakistan Med Assoc. 2017;67(4):544. 28420912

[10] Drakonaki E, Chatziioannou M, Spiridakis K, Panagiotakis G Acute abdomen caused by a small bowel perforation due to a clinically unsuspected fish bone. Diagn Interv Radiol. 2011;17(2):160 10.4261/1305-3825.DIR.3236-09.1 20683816

[11] Goh BK, Chow PK, Quah HM, et al Perforation of the gastrointestinal tract secondary to ingestion of foreign bodies. World J Surg. 2006;30(3):372–377. 10.1007/s00268-005-0490-2 16479337

[12] Goh BK, Jeyaraj PR, Chan HS, et al A case of fish bone perforation of the stomach mimicking a locally advanced pancreatic carcinoma. Dig Dis Sci. 2004;49(11/12):1935–1937. 10.1007/s10620-004-9595-y 15628728

[13] Bajwa A, Seth H, Hughes F Ingested fishbone mimicking a gastric submucosal tumour. Grand Rounds. 2007;7:42–44. 10.1102/1470-5206.2007.0014

[14] Beecher SM, O’Leary DP, McLaughlin R Diagnostic dilemmas due to fish bone ingestion: Case report & literature review. Int J Surg Case Rep. 2015;13:112–115. 10.1016/j.ijscr.2015.06.034 26188981 PMC4529669

